# Rapid diagnostic work-up of *Streptococcus dysgalactiae* endophthalmitis by a novel culture processing system

**DOI:** 10.1099/acmi.0.000645.v3

**Published:** 2023-11-21

**Authors:** Erin Su, Jessica Chang, Khay Ugaban, Rosemary C. She

**Affiliations:** ^1^​ Department of Ophthalmology, Keck School of Medicine of the University of Southern California, Los Angeles, CA, USA; ^2^​ Keck Medical Center of the University of Southern California, Los Angeles, CA, USA; ^3^​ Department of Pathology, Keck School of Medicine of the University of Southern California, Los Angeles, CA, USA

**Keywords:** vitreous fluid, rapid diagnostics, *Streptococcus*

## Abstract

The diagnosis of infective endophthalmitis is supported by microbiological work-up. Rapid work-up is critical to confirm clinical suspicion and appropriate antimicrobial therapy. We report the novel use of an automated liquid culture processing system (FAST system, Qvella, ON, Canada) in a vitreous fluid culture. A 59-year-old patient with post-operative endophthalmitis presented with acute right eye pain and blurred vision. Vitreous fluid collected for microbiology culture was of limited quantity and only inoculated to thioglycolate broth. The broth recovered beta-haemolytic, group G *Streptococcus dysgalactiae* susceptible to penicillin and vancomycin. Experimental application of the FAST system to purify the organism from broth culture yielded the same identification and susceptibility test results but 1 day sooner. Despite prompt treatment with appropriate antibiotics, including vancomycin and ceftazidime, disease progressed rapidly and required enucleation to achieve a stable therapeutic outcome. Use of automated processing of monomicrobial broth cultures has thus far focused on positive blood culture broths, but could potentially include other liquid-based cultures such as for sterile body fluids of critical nature.

## Data Summary

No data were generated or reused.

## Introduction

Endophthalmitis typically presents with pain and decreased vision and often results in permanent blindness and may require surgical intervention. One decisive prognostic factor in endophthalmitis is the triggering pathogen [1]. *Candida* spp. or coagulase-negative staphylococci typically have favourable prognoses, while *Bacillus *, *Aspergillus* and *Streptococcus* spp. have unfavourable prognoses. In such urgent cases, which may result in orbital cellulitis, efficient and accurate diagnosis is key in patient treatment and prognosis.

Bacterial cultures of the vitreous fluid are routinely performed in such cases [2]. Broth cultures, either with conventional thioglycolate broth or blood culture bottles, increase the yield of pathogen recovery compared to solid media alone [3, 4]. Although broth cultures offer added sensitivity, direct analysis of growth is largely limited to the Gram stain, while definitive identification and antimicrobial susceptibility testing (AST) rely on subcultured growth on solid media. This added step extends the work-up by the time required for colony growth to become visible, usually one or more days. Here, we report the novel use of a recently developed liquid processing system (FAST system, Qvella, ON, Canada), which purifies organisms growing in liquid culture media for direct downstream application in identification and susceptibility testing. Applying the system to a vitreous fluid culture from a case of exogenous bacterial endophthalmitis, we demonstrate its potential to accelerate the work-up of critical pathogens from broth media.

## Case presentation

A 59-year-old man presented with severe, sudden-onset right eye pain and decreased vision in the setting of multiple surgical interventions of the orbit within the past few years and recurrent bilateral anterior uveitis. Previous ophthalmic procedures included bilateral primary open-angle glaucoma with glaucoma drainage devices implanted in both eyes, cataract extraction and intraocular lens insertion, recurrent bilateral anterior uveitis, pars plana vitrectomy and bilateral scleral buckles. Medications included prednisolone and lifitegrast eye drops, oral prednisone and oral valacyclovir. Other examination findings upon presentation included chemosis, corneal oedema, and vitreous inflammation and consolidation with inferior scleral and choroidal thickening. The patient also showed orbital oedema, erythema and lid swelling from concurrent preseptal cellulitis.

Within 24 h of symptom onset, the right eye underwent vitreous tap, anterior chamber paracentesis and intravitreal injection of vancomycin, ceftazidime and foscarnet. The vitreous fluid was observed to be clear and non-turbid and was submitted for bacterial and fungal cultures. Only thioglycolate broth was inoculated for bacterial cultures due to minimal total sample volume (<0.3 ml). Direct specimen Gram stain revealed four plus Gram-positive cocci in chains and no white blood cells. After overnight incubation, the thioglycolate broth demonstrated turbid growth in the mid to lower zones, with Gram stain of the broth growth showing Gram-positive cocci in chains. The provider requested urgent organism identification, at which point the laboratory was only able to advise that findings were consistent with *Streptococcus* or *Enterococcus* spp. Per standard workflow, subcultures of the broth were made to blood and chocolate agar for aerobic culture and *Brucella* agar for anaerobic culture. After overnight incubation, beta-haemolytic colonies were isolated and identified as group G *Streptococcus* sp. by streptococcal typing (PathoDx, Remel, Lenexa, KY), and as *Streptococcus dysgalactiae* ssp. *dysgalactiae/S. dysgalactiae* ssp. *equisimilis* by matrix-assisted laser desorption/ionization time-of-flight (MALDI-TOF) mass spectrometry (Vitek MS v3.2, bioMérieux, Lyon, France). AST conducted by E-test (bioMérieux) resulted in minimal inhibitory concentrations (MICs) of 0.023 µg ml^−1^ for penicillin (susceptible) and 0.19 µg ml^−1^ for vancomycin (susceptible). All other infectious disease testing was negative, including PCR of anterior chamber fluid for herpes simplex virus 1 (HSV-1), herpes simplex virus 2 (HSV-2), varicella-zoster virus (VZV), cytomegalovirus (CMV) and *Toxoplasma gondii*, vitreous fluid fungal culture and serological testing (RPR, *Toxoplasma* IgG and IgM, *Borrelia* IgG and IgM, and interferon-gamma release assay for tuberculosis).

For investigative purposes (the results of which were not clinically reported), at first appearance of growth, 2 ml of the thioglycolate broth was processed on the FAST system (Qvella, ON, Canada) using the manufacturer’s instructions. The system consists of a processor and disposable cartridge that concentrate and purify micro-organisms from liquid media with a runtime of ~20 min. The resulting isolate suspension was adjusted to 0.5 McFarland in half-normal saline for all downstream work-up. Identification was performed by Vitek MS MALDI-TOF by spotting 1 µl organism suspension to each of four wells of the target plate. This was immediately followed by formic acid extraction and acquisition of protein spectra per the manufacturer’s instructions. The organism was identified with high confidence as *Streptococcus dysgalactiae* ssp. *dysgalactiae/Streptococcus dysgalactiae* ssp. *equisimilis*. Streptococcal typing (PathoDx) was positive for group G and negative for groups A, B, C and F. AST by E-test for penicillin and vancomycin were 0.032 µg ml^−1^ for penicillin (susceptible) and 0.38 µg ml^−1^ for vancomycin (susceptible). By comparison to the standard workflow, the investigative FAST system allowed accurate identification and AST results to be obtained 1 day earlier (Table 1).

**Table 1.** Summary of standard of care (SOC) compared to investigative results using FAST system liquid colony for identification and AST. Days to result indicates time from initial specimen planting

Test	Result	Days to result
SOC identification, MALDI-TOF, subcultured colony Liquid colony identification, MALDI-TOF	* Streptococcus dysgalactiae* ssp. *dysgalactiae/S. dysgalactiae* ssp. *equisimilis* (50/50%) * Streptococcus dysgalactiae* ssp. *dysgalactiae/S. dysgalactiae* ssp. *equisimilis* (50/50%)	2 1
SOC vancomycin E-test Liquid colony vancomycin E-test	0.19 µg ml^−1^ (susceptible) 0.38 µg ml^−1^ (susceptible)	3 2
SOC penicillin E-test Liquid colony penicillin E-test	0.023 µg ml^−1^ (susceptible) 0.032 µg ml^−1^ (susceptible)	3 2

In addition to intravitreal antibiotics, the patient was treated for exogenous endophthalmitis as an outpatient with gatifloxacin eye drops and oral moxifloxacin, and for pre-septal cellulitis with oral amoxicillin/clavulanate. Following AST results, another intravitreal dose of vancomycin was given, and fortified vancomycin eye drops were prescribed to specifically treat streptococcal endophthalmitis. Despite timely and appropriate medical management, right eye pain and vision loss progressed. Six days after initial presentation, enucleation was performed due to concern for the cellulitis progressing to orbital infection. Histopathological evaluation of the right eye confirmed acute endophthalmitis with suppurative inflammation noted to involve the fibrous capsule, cornea, anterior chamber, vitreous, iris, ciliary epithelium and retina. Other findings included intraretinal haemorrhages and exudates, but no bacterial or fungal organisms were seen on Gram, GMS, or PAS stains. Two weeks after enucleation, a secondary implant was placed, with successful implant coverage and patient recovery.

## Discussion

Given the patient’s extensive ocular and surgical history, there were multiple opportunities for complications that could have led to endophthalmitis. Most notable of these include multiple previous episodes of bilateral anterior, herpetic uveitis, prior ocular procedures, and the patient’s immunosuppressive corticosteroid medications. The sudden onset of the patient’s symptoms was suggestive of an acute infectious aetiology, the sources of which are often posttraumatic or postoperative [1]. However, the patient had no prior history of ocular trauma, and his most recent eye operation was 6 weeks prior to endophthalmitis symptom onset and was performed in the other eye. The most recent operation in the affected eye was 18 months prior to symptom onset, with no other signs of acute or indolent infection in the interim. Taking these factors in consideration and the lack of other systemic infections or indwelling catheters or other devices, the infection was most likely bleb-related endophthalmitis. Given the overlying hardware of the previously placed glaucoma shunt, the conjunctiva likely thinned over time, allowing the spread of surface bacteria. Aggressive bleb-associated endophthalmitis cases have been shown to occur up to several years after glaucoma filtering surgery, with cultures often positive for *Streptococcus* spp. and with similar, acute presentations [5]. The rapid progression of disease and preseptal spread in this case were likely facilitated by the glaucoma implant shunt system already in place that allowed communication between the intraocular space to the subconjunctival space and therefore bacterial spillover.

*S. dysgalactiae* is a recognized but rarely reported cause of endophthalmitis [6]. Of note, longitudinal studies specifically examining streptococcal endophthalmitis found viridans group streptococci to be the most common cause but did not identify any *S. dysgalactiae* in their reviews [7, 8]. Endogenous cases of *S. dysgalactiae* endophthalmitis originating from infective endocarditis, meningitis and aortic root abscess, as well as posttraumatic exogenous cases, have been reported. Outcomes varied from complete recovery to rapid progression and permanent visual impairment despite appropriate antibiotic treatment [6–13]. Our case reinforces the potential of this organism to cause rapid, fulminant ophthalmic infections. Even though vancomycin and a third-generation cephalosporin were administered to treat a susceptible organism at the point of care, enucleation was required for patient recovery.

Diagnosis of endophthalmitis is primarily clinical, but microbiological cultures and vitreous or anterior chamber PCR results offer strong support and may direct antimicrobial treatment regimens [1]. Efficient and reliable diagnostic tools are helpful in early intervention. In this case, clinical urgency required rapid provision of culture results. While the laboratory’s suspicion for *Streptococcus* spp. was strong based on direct specimen Gram stain findings and pattern of growth in thioglycolate broth, *Enterococcus* spp., which have a different antimicrobial resistance profile, could not be ruled out. Species identification was only possible with definitive testing of the organism isolate. Standard workflow requires use of colonies grown on solid media for identification and susceptibility testing.

The recently developed FAST system, used here on an experimental basis, allows identification and AST to be performed directly from broth cultures by removing cellular materials, proteins and other interfering substances that may be found in broth. Intended for automated processing of blood culture specimens, the FAST system has yielded promising results in early studies to support clinical use when paired with various downstream identification and AST platforms [13–17]. Prospective studies have found that for positive blood cultures, FAST system processing can be used to rapidly obtain correct organism identifications by MALDI-TOF in 87–96 % of cases, and AST results with ~98 % categorical agreement with standard of care AST [13–18]. Not only has the FAST system been shown to be highly accurate, it has also been shown by other studies to significantly reduce workflow time [17, 18].

A rapid specimen processor such as the FAST system could theoretically be applied to other types of broth cultures with monomicrobial growth, not only blood cultures, as illustrated in our case. While manual processes exist to purify organism isolates from broth for immediate use, they are typically labour intensive, involving steps such as serial wash–spin cycles that preclude them from widespread adoption [19–21]. The same is true for commercial kits such as the Sepsityper (Bruker Daltonics, Billerica, MA, USA), which is designed for direct from broth organism identification by MALDI-TOF, but not for phenotypic AST [19]. Broth cultures are routinely set up by the clinical laboratory on critical specimen types such as sterile body fluids and invasively collected tissues; therefore, an accelerated work-up could positively influence patient treatment and management. In the case of vitreous fluids, broth cultures play a key role in increasing the pathogen yield over solid media alone. Blood culture bottle systems furthermore provide optimal recovery compared to conventional thioglycolate broth [4]. The frequency of polymicrobial infection in endophthalmitis is low, <5 % in several studies [3, 4], thus facilitating application of rapid processing such as with the FAST system. Certainly, a careful and thorough approach to evaluating novel instruments is warranted prior to clinical use for off-label indications, and studies are ongoing in our laboratory to fully explore the potential.

In summary, we report an unusual case of exogenous endophthalmitis caused by beta-haemolytic, group G *Streptococcus dysgalactiae *. We found that with a specialized liquid processing system, a precise identification and AST results could be obtained 1 day faster from a broth culture compared to standard workflow. Although the results likely would not have significantly altered the clinical course in the presented case, we demonstrate the potential of a rapid approach towards culture work-up from a critical specimen source.
